# Sensor-Based Reachable Workspace Assessment for Tracking Upper-Extremity Mobility Changes over Time Following the Spencer Technique in Older Adults

**DOI:** 10.3390/s26196305

**Published:** 2026-10-06

**Authors:** Vicky Chan, Andria Farrens, Nathan Han, Addy Chen, Hannah Nguyen, Juan Carlos Perez-Ibarra, Richard Thai, Jason Koh, Jay J. Han

**Affiliations:** 1 Department of Physical Medicine and Rehabilitation, University of California at Irvine School of Medicine, Irvine, CA 92868, USA; nathan.han.27@stu.creanlutheran.org (N.H.); 28chena@sagehillschool.org (A.C.); 27nguyenhannah@iusd.org (H.N.); jperezib@hs.uci.edu (J.C.P.-I.); jayjhan@hs.uci.edu (J.J.H.); 2 Department of Rehabilitation Services, Irvine Medical Center, University of California, Orange, CA 92868, USA; 3 Research Institute at Children Hospital Orange County (CHOC), Orange, CA 92868, USA; andria.farrens@choc.org; 4 Veterans Affairs Hospital in Long Beach (VALB), Long Beach, CA 90822, USA; richard.thai@va.gov; 5 MemorialCare Rehabilitation Institute, MemorialCare Long Beach Medical Center, Long Beach, CA 90806, USA; jkoh@memorialcare.org

**Keywords:** reachable workspace, range of motion, elderly, upper extremity, Kinect

## Abstract

Age-related declines in upper-extremity mobility remain a challenge to assess in older adults. This study examined whether a sensor-based motion analysis of reachable workspace (RWS), quantified using relative surface area (RSA), could track longitudinal upper-extremity mobility changes in a cohort of community-dwelling older adults receiving the Spencer Technique, an established therapeutic intervention for the shoulder. Fifteen community-dwelling older adults ≥65 years with shoulder mobility limitations identified by reduced goniometric ROM received the Spencer Technique twice weekly for two weeks. Outcomes including RSA, shoulder flexion and abduction via goniometry, Apley scratch test, DASH and upper extremity NeuroQoL were assessed at baseline, weekly, post-therapy, and 1-month follow-up. Total RSA significantly increased from baseline to post-therapy and 1-month follow-up in both dominant and nondominant upper extremities and remained significant after Holm correction (adjusted *p* ≤ 0.030). Bilateral shoulder flexion and abduction along with Apley scratch tests demonstrated parallel improvements. DASH scores did not demonstrate significant change, whereas NeuroQoL UE scores increased significantly at 1-month follow-up after Holm correction (adjusted *p* = 0.031). Total RSA demonstrated responsiveness to longitudinal changes in upper-extremity reachability following the Spencer Technique that parallel changes observed with standard clinical assessments. These findings support the utility of sensor-based RWS as a quantitative measure for tracking longitudinal upper-extremity mobility over time in older adults.

## 1. Introduction

The aging population may experience age-related declines in upper-extremity range of motion that can significantly impact activities of daily living (ADLs). According to the 2022 U.S. Census report, 13.4% of the U.S. civilian noninstitutionalized population, 44.1 million people, reported having a disability. Of this population, 15.4 million had independent living difficulty, with 7.5 million adults experiencing self-care difficulty [1]. The U.S. population aged 65 years and older is projected to increase from approximately 40.2 million in 2010 to 88.5 million by 2050, representing approximately 20.2% of the total U.S. population [2]. These disabilities and limitations often impair basic self-care activities at home, including feeding, grooming, dressing, and toileting.

Upper-extremity impairment accompanies numerous neurological, neuromuscular, and musculoskeletal conditions (e.g., stroke, traumatic brain injury, arthritis, muscular dystrophy). Although these conditions can produce recognizable patterns of impairment, reduced upper-extremity mobility may also occur in older adults without a specific diagnosed neurological or musculoskeletal disorder. Individuals aged 65 and older with a single chronic disease are twice as likely to need support for basic activities of daily living (ADLs), whereas those with two or more chronic conditions face over a fourfold increase in risk. As the older adult population continues to grow, identifying and addressing age-related functional limitations, particularly upper-extremity-associated disabilities that result in functional dependency, will become increasingly important.

Aging is associated with progressive changes in musculoskeletal flexibility and joint mobility. Reduced shoulder mobility resulting from decreased flexibility, rotator cuff issues or shoulder instability may interfere with the complex movements required for daily activities. Longitudinal studies have demonstrated age-related declines in flexibility among older adults [3], while age-related alterations in collagen structure, crosslinking, and turnover may contribute to changes in tissue mechanical properties [4]. Shoulder symptoms and mobility limitations are also common in older adults and may adversely affect performance of daily activities [5]. Consequently, effectively addressing and managing upper-extremity disabilities is essential for enhancing the quality of life within this expanding demographic. Identifying subtle changes in upper-extremity function and providing therapeutic intervention becomes increasingly important.

Traditional evaluation of shoulder mobility and function relies on clinician-based assessments such as goniometric ROM. These established measures provide useful objective information about shoulder movement but are limited when assessing multidirectional upper-extremity movement required for functional tasks, require trained professionals to administer, and take 10–20 min to complete. Patient-reported outcome (PRO) measures such as the Quality of Life in Neurological Disorders Upper Extremity scale (NeuroQoL UE) and the Disabilities of the Arm, Shoulder and Hand (DASH) questionnaire provide complementary information regarding patient-perceived difficulty with upper-extremity activities but reflect subjective perceptions of function that vary across patients [6,7]. Objective measures characterizing functional upper-extremity ROM may complement traditional assessments of range of motion and PROs in the evaluation of age-related upper-extremity impairment and response to treatment.

Sensor-based motion analysis can provide objective measures of upper-extremity movement. Our group has developed the reachable workspace (RWS) assessment, which measures the three-dimensional surface area generated as an individual performs a standardized multidirectional upper-extremity reaching protocol. RWS is quantified using relative surface area (RSA), which normalizes reachable surface area to the participant’s arm length to enable comparisons across individuals [8,9,10,11,12]. RWS integrates movement across multiple directions and provides a quantitative and spatial representation of upper-extremity reachability.

The validity, reliability and sensitivity of sensor-derived RWS have been evaluated across populations with upper-extremity impairment, including individuals with neurological, neuromuscular, and musculoskeletal conditions [8,9,10,11,12,13]. Our previous research demonstrated the ability of RSA to identify previously undiagnosed limitations in the upper-extremity range of motion in older adults [9]. However, the responsiveness of RSA to longitudinal changes following therapeutic intervention in community-dwelling older individuals has not been established.

Here, we tested whether sensor-based RWS measures complement conventional clinical measures when monitoring therapeutic changes over time, using the established Spencer Technique for the shoulder. The Spencer Technique is a standardized osteopathic manipulative intervention for the mobilization of the glenohumeral and scapulothoracic joints to increase ROM at the shoulder. Prior randomized studies have established the therapeutic effect of the Spencer Technique in patients with pre-existing shoulder impairment on ROM and functional ability [14]. A recent systematic review and meta-analysis with 16 randomized controlled trials involving 659 patients found the Spencer Technique reduced pain and disability and improved ROM [15]. The Spencer Technique therefore provides a standardized therapeutic intervention through which to evaluate the responsiveness of sensor-based RWS to longitudinal changes in shoulder mobility.

The primary objective of this proof-of-concept study was to determine whether sensor-based RWS, quantified using RSA, could detect longitudinal changes in upper-extremity mobility in community-dwelling older individuals following therapeutic treatment of the shoulder with the Spencer Technique. Shoulder ROM as measured by goniometry, functional shoulder mobility by Apley test and PROs including DASH and NeuroQoL UE were included as complementary clinical outcomes. The primary comparisons were changes from baseline to immediately post-therapy and from baseline to 1-month follow-up. We hypothesized that Total RSA would increase from baseline to post-therapy and that these changes would be maintained at 1-month follow-up, supporting the responsiveness of sensor-based RWS for tracking longitudinal upper-extremity mobility.

## 2. Methods

### 2.1. Study Design

This proof-of-concept, single-group longitudinal study evaluated whether sensor-based RWS, quantified by RSA, could track longitudinal changes in upper-extremity shoulder mobility over time in a population of community-dwelling older adults with decreased shoulder range of motion after they received the Spencer Technique.

This study recruited participants from a previous cross-sectional study of 61 community-dwelling adults aged ≥65 who underwent upper-extremity ROM and RWS assessments [9]. From this cohort, 15 individuals were identified as having reduced shoulder mobility, defined as having at least a 10% reduction in active shoulder flexion or abduction compared with the previously defined mean ROM for an age-matched reference group [16]. The remaining 46 participants contributed to the baseline data. Since this group did not undergo intervention or longitudinal follow-up, they were not considered an untreated control group. Longitudinal analysis was restricted to within-participant changes in the 15 participants.

The study cohort underwent four assessments: baseline, weekly, post-therapy, and 1-month follow-up. Participants received four 30 min intervention sessions conducted twice weekly over 2 weeks. The baseline assessment was conducted at a separate visit before the first treatment session. The weekly assessment was performed following the second treatment session, and post-therapy was conducted at a separate visit within 3 days after completion of the fourth treatment session. The final assessment was conducted at 1-month follow-up. The weekly assessment was used to characterize change during intervention, whereas post-therapy and 1-month follow-up were the primary longitudinal endpoints for comparison with baseline. The study consisted of 7 visits in total.

### 2.2. Participants

Fifteen individuals met the inclusion criteria for reduced shoulder mobility and were enrolled in the study. No participants were excluded after application of the exclusion criteria. All 15 participants reported right-hand dominance and completed all longitudinal assessments (Figure 1).

#### 2.2.1. Inclusion Criteria

Participants were eligible for this longitudinal study if they: (1) were aged ≥65 years, (2) had participated in our previous RWS cross-sectional study, and (3) demonstrated at least a 10% reduction in active shoulder ROM as compared to a previously defined mean for an age-matched reference group.

#### 2.2.2. Exclusion Criteria

Participants were excluded if they had: (1) a confirmed diagnosis of a severe neurological, musculoskeletal, or neuromuscular condition; (2) significant spasticity, contractures, ataxia, tremor, limb deformity, or movement disorder, or severe cognitive impairment; (3) an inability to follow study directions; or (4) planned surgery or major procedure involving the upper extremity, brain, or cervical spine within 6 months following enrollment.

### 2.3. Sample Size Determination

The sample size was based on the eligible participants identified from the previously characterized cohort and the anticipated within-participant improvement in active shoulder ROM. We expected that participants would improve within-participant upper-extremity goniometric measurements from baseline to 2 weeks post-treatment by a mean of 10 degrees (SD = 10 degrees) and a sample of 15 participants provided approximately 93% power to test this hypothesis at the 5% significance level using a two-sided paired *t*-test.

The original sample-size calculation was based on goniometric ROM rather than the sensor-derived RSA outcome. Therefore, the present study should be considered a proof-of-concept evaluation of longitudinal RSA change rather than a definitively powered evaluation of RSA responsiveness or treatment efficacy.

### 2.4. Ethical Review

This study protocol was approved by the University of California, Irvine Institutional Review Board (IRB) number HS# 2017-3801, and written informed consent was obtained prior to the start of the study procedures.

### 2.5. Bias

To minimize selection bias, consecutive participants meeting the inclusion criteria were enrolled. To promote consistency of study procedures, the Spencer Technique was administered by a single osteopathic physician, and all clinical assessments were performed by a single therapist evaluator.

RWS assessment procedures were standardized across all visits. Prior to each RWS assessment, participants viewed the same instructional video and completed a practice trial. The evaluator confirmed correct performance of the standardized movement sequence, verified adequate sensor tracking, and monitored for compensatory movement patterns. Verbal corrections and additional instructions were provided as needed before the recorded assessment.

All recorded RSA data were subsequently processed by an independent engineer who was not involved in the clinical visits, intervention delivery, or clinical outcome assessments.

### 2.6. Setting

Participants from the previous study were invited back for this study from August 2019 to November 2019. Medical history, demographic, anthropometric information (including age, gender, height, and weight) and clinical measures were obtained at the baseline evaluation.

### 2.7. Spencer Technique

Participants received the Spencer Technique in an outpatient clinic at UC Irvine Health Center. The Spencer Technique was developed in 1915 by Charles H. Spencer, D.O., as an articulatory technique to increase ROM at the shoulder. The seven-step protocol for mobilization of the glenohumeral and scapulothoracic joints included (1) extension with elbow flexed; (2) flexion with elbow extended; (3) compression circumduction; (4) circumduction with traction with elbow flexed; (5) adduction with external rotation with elbow flexed; (6) internal rotation; and (7) stretching tissues and pumping fluids with the arm extended [17]. Each treatment session lasted approximately 30 min.

### 2.8. Clinical Outcomes

#### 2.8.1. Goniometric Measurements of Shoulder Motion in Flexion and Abduction

Active ranges of motion in shoulder flexion and abduction were measured by a universal goniometer. Although the recommended position in testing shoulder flexion and abduction is in supine [18], this study tested all study participants in a sitting position to be consistent with testing position for the upper extremity reachable workspace. Sabari et al. [19] reported high intra-rater reliability for active measurements of shoulder flexion and abduction (ICC 0.97 and 0.97, respectively) regardless of whether testing is administered with subjects in the supine or sitting positions, as long as testing was administered in a consistent position. Shoulder flexion and abduction were measured in degrees (°). All testing was performed by a single tester throughout the study.

#### 2.8.2. Upper Extremity Reachable Workspace Protocol and Analysis

The upper extremity reachable workspace protocol measured by the Microsoft Kinect 2.0 sensor (Redmond, WA) followed the previously published protocol [9,10,11,12]. The Kinect sensor acquired color and depth data at 30 frames per second (fps), consistent with the previously published RWS methodology [8]. The Kinect sensor was positioned 230 cm from the participant, at a height of 115 cm. All study participants were seated in front of the Microsoft Kinect sensor and viewed an instructional video demonstrating the movement protocol and performed standardized upper-extremity movements. Dominant and nondominant upper extremities were assessed separately. Each participant completed a practice trial during which the evaluator confirmed correct performance of the standard movement protocol. Following the practice trial, participants completed one recorded RWS assessment for each arm (Figure 2A).

With the elbow maintained in extension, participants raised the tested arm from the resting position to above the head through standardized vertical planes at approximately 0°, 45°, 90°, and 135°. The movement sequence incorporated shoulder abduction, scaption, flexion, horizontal abduction and adduction, and extension and adduction. Participants subsequently performed horizontal sweeping movements approximately at the level of the umbilicus and shoulder. The standardized RWS movement sequence required approximately 1 min per arm. During the recorded assessment, the evaluator continued to monitor movement performance and sensor tracking to ensure adherence to the standardized protocol.

For analysis, the reachable workspace envelope was split into five quadrants (upper medial and lateral (Q1, Q3), lower medial and lateral (Q2, Q4), and posterior inferior-lateral (Q5)) with the shoulder joint serving as the origin (Figure 2B,C). In order to allow for comparison between patients, the absolute total and quadrant reachable workspace surface envelope areas (m^2^) were normalized by each individual’s arm length (r) to obtain the relative surface area (RSA). This RSA represents the portion of a frontal unit hemisphere and is determined by dividing the measured absolute reachable workspace area by the factor 4πr^2^ × (1/2). The four frontal quadrants (Q1–Q4) collectively represent the frontal hemispheric workspace, whereas Q5 represents the additional posterior inferior–lateral reachable workspace [12].

Total RSA and quadrant-specific RSA (Q1–Q5) were calculated separately for the dominant and non-dominant upper extremities. All RWS data were processed by a single engineer using MATLAB (version 9.13.0, R2022b; The MathWorks, Inc., Natick, MA, USA). Skeletal tracking, reachable-surface reconstruction, and RSA computation were performed using the previously published RWS processing methodology [8,9,10,11,12]. The same processing procedures were applied across all participants and assessment time points.

#### 2.8.3. NeuroQoL

The Quality of Life in Neurological Disorders (NeuroQoL) is a 20-question self-reported assessment for adults and children with neurologic disorders. The assessment comprises item banks and scales evaluating symptoms, concerns and issues for the ability to carry out various activities involving digital, manual and reach-related functions, ranging from fine motor to self-care tasks associated with activities of daily living. We used the assessment related to impairments of the upper extremity. Participants were given a list of items related to the upper extremity and asked about the ability to perform these tasks: without any difficulty, with a little difficulty, with some difficulty, with much difficulty, or unable to do. Each response is recorded on the score sheet and is assigned a number within the range of 1–5. These numbers determine the raw score. The range of scores is 20–100, with the higher score indicating less impairment [6].

#### 2.8.4. DASH

The Disabilities of the Arm, Shoulder and Hand (DASH) survey is a 30-item self-reported questionnaire designed to assess musculoskeletal disorders of the upper extremities. The survey has two 4-item modules used to measure symptoms and functions. The participant rates their ability to do the activities in the last week by circling the number corresponding to the appropriate response. Item responses range from 1 (no difficulty, not at all, strongly disagree) to 5 (unable, extremely, strongly agree). Lower scores indicate a better outcome [7].

#### 2.8.5. Apley Scratch Test

This test evaluates shoulder mobility and range of motion by assessing the participant’s ability to reach the opposite shoulder and back. Each movement is performed bilaterally and involves two movements: one arm reaches behind the head to touch the superior portion of the opposite scapula, while the other arm reaches behind the back to touch the inferior portion of the opposite scapula. In this study, Apley 1 refers to extension, internal rotation, and adduction of the dominant arm along with flexion, external rotation and abduction of the nondominant arm. Apley 2 refers to flexion, external rotation, and abduction of the dominant arm and extension, internal rotation, and adduction of the nondominant arm. Three trials are conducted for each movement, during which the distance in centimeters between the middle fingers of each hand is recorded. The average of the three trials was used for analysis [20].

### 2.9. Statistical Analysis

The demographic and baseline clinical characteristics were summarized using the mean and standard deviation (SD) for continuous variables and the frequency and percentage for categorical variables. Baseline characteristics of participants with and without reduced shoulder mobility were compared using independent-sample *t*-tests. The 46-participant baseline comparison cohort was used only to characterize cross-sectional normative values and was not included as a longitudinal control group.

Longitudinal changes among the 15 study participants were evaluated using repeated-measures linear mixed models. Time was modeled as a categorical fixed effect with four levels (baseline, weekly, post-therapy, and 1-month follow-up), with the participant specified as the subject variable and a compound-symmetry covariance structure used to account for within-participant repeated measurements. Models were estimated using restricted maximum likelihood with Satterthwaite denominator degrees of freedom.

The principal longitudinal comparisons were baseline to post-therapy and baseline to 1-month follow-up. Weekly assessment was included in the overall longitudinal models to characterize the trajectory of change but was not included among the planned endpoint comparisons subjected to multiplicity correction. To control for multiple comparisons, raw *p*-values were adjusted using the Holm procedure separately across related outcome measures [21]: Total RSA, clinical ROM, Apley, patient-reported outcomes, and exploratory quadrant-specific RSA measures. Estimated mean changes with 95% confidence intervals (CIs), raw and Holm-adjusted *p*-values, and within-participant effect sizes, using Cohen’s *dz,* defined as the mean paired change divided by the standard deviation of the individual change scores, were reported. Statistical significance for the planned endpoint comparisons was defined as Holm-adjusted *p* < 0.05. Analyses were performed using IBM SPSS Statistics, version 29 (SPSS Inc., Chicago, IL, USA).

## 3. Results

### 3.1. Study Participant Characteristics

Of the 61 community-dwelling older adults in the previously characterized cohort, a total of 15 participants were included in the longitudinal cohort to undergo the Spencer Technique. The mean age was 75.53 ± 5.48 years, and six participants (40%) were male. When comparing the 15 participants identified as having reduced shoulder mobility to the baseline cohort, no significant differences were observed in age, height, or weight. There were significant between-group differences in goniometric measurements of shoulder range of motion. Specifically, significant differences were observed in dominant shoulder flexion (*p* = 0.002) and abduction (*p* < 0.001), as well as in nondominant shoulder flexion (*p* < 0.001) and abduction (*p* < 0.001). DASH (*p* = 0.041) and NeuroQoL UE (*p* = 0.038) were also significantly different between the two groups (Table 1).

### 3.2. Reachable Workspace (RSA)

The study group was evaluated with a motion capture device (Kinect) measuring the total reachability quantified, using RSA in a seated position. The RWS envelope was divided into five separate quadrants with four in the frontal plane (Figure 2). Descriptive RSA values across assessment time points are presented in Table 2, with a representative participant visualization shown in Figure 3. 

Total RSA demonstrated significant overall changes over time in both the dominant arm [*F*(3,42) = 7.62, *p* < 0.001] and non-dominant arm [*F*(3,42) = 3.35, *p* = 0.028]. In the dominant arm, the Total RSA increased by 0.090 from baseline to post-therapy (95% CI, 0.036 to 0.143; Holm-adjusted *p* = 0.005; Cohen’s *dz* = 0.74) and by 0.119 from baseline to 1-month follow-up (95% CI, 0.065 to 0.172; Holm-adjusted *p* < 0.001; Cohen’s *dz* = 0.88).

In the nondominant arm, Total RSA increased by 0.050 from baseline to post-therapy (95% CI, 0.005 to 0.096; Holm-adjusted *p* = 0.030; Cohen’s *dz* = 0.79) and by 0.068 at 1-month follow-up (95% CI, 0.023 to 0.114; Holm-adjusted *p* = 0.008; Cohen’s *dz* = 0.65) (Table 3 and Figure 4).

Quadrant-specific changes were less consistent. No quadrant-specific post-therapy comparison remained significant after Holm correction, and only dominant Q1 at 1-month follow-up (mean change = 0.038; Holm-adjusted *p* = 0.046) and nondominant Q5 at 1-month follow-up (mean change = 0.022; Holm-adjusted *p* = 0.013) remained statistically significant (Supplementary Table S1).

### 3.3. Range of Motion (ROM)

Table 4 and Figure 5 summarize goniometric shoulder ROM measurements of all participants at baseline, weekly, post-therapy and 1-month follow-up.

Significant overall time effects were observed for dominant flexion [*F* (3,42) = 6.91, *p* < 0.001], dominant abduction [*F* (3,42) = 9.78, *p* < 0.001], nondominant flexion [*F* (3,42) = 11.07, *p* < 0.001], and nondominant abduction [*F* (3,42) = 17.14, *p* < 0.001]. All eight planned baseline-to-post-therapy and baseline-to-1-month comparisons remained statistically significant after Holm correction.

Dominant flexion increased by 17.0° post-therapy (95% CI, 8.04° to 25.97°; Holm-adjusted *p* < 0.001; Cohen’s *dz* = 0.72) and by 17.5° at 1-month follow-up (95% CI, 8.50° to 26.43°; Holm-adjusted *p* < 0.001; Cohen’s *dz* = 0.70). Dominant abduction increased by 21.7° at post-therapy (95% CI, 11.91° to 31.55°; Holm-adjusted *p* < 0.001; Cohen’s *dz* = 0.90) and by 23.5° at 1-month follow-up (95% CI, 13.71° to 33.35°; Holm-adjusted *p* < 0.001; Cohen’s *dz* = 0.82).

Nondominant flexion increased by 13.1° at post-therapy (95% CI, 8.23° to 18.03°; Holm-adjusted *p* < 0.001; Cohen’s *dz* = 1.17) and by 10.4° at 1-month follow-up (95% CI, 5.50° to 15.30°; Holm-adjusted *p* < 0.001; Cohen’s *dz* = 0.96). Nondominant abduction increased by 17.4° at post-therapy (95% CI, 11.8° to 23.0°; Holm-adjusted *p* < 0.001; Cohen’s *dz* = 2.14) and by 17.1° at 1-month follow-up (95% CI, 11.5° to 22.7°; Holm-adjusted *p* < 0.001; Cohen’s *dz* = 1.33) (Table 5).

### 3.4. Apley Scratch Test

Both Apley scratch test measures demonstrated significant changes over time, with improvements maintained at 1-month follow-up. All four planned comparisons remained significant after Holm correction. Apley 1 inter-finger distance decreased by 4.50 cm at post-therapy and by 4.48 cm at 1-month follow-up, while Apley 2 decreased by approximately 4.69 cm and 5.29 cm, respectively. Smaller inter-finger distances represent greater combined shoulder mobility. Effect sizes and 95% CIs are presented in Table 6 and Table 7.

### 3.5. Patient-Reported Outcomes (PRO)

Patient-reported outcomes demonstrated less consistent changes than the objective mobility measures. NeuroQoL UE increased by 2.0 points from baseline to post-therapy and by 3.4 points from baseline to 1-month follow-up. Only the 1-month comparison remained statistically significant after Holm correction (Holm-adjusted *p* = 0.031). The post-therapy NeuroQoL change was not significant after multiplicity correction (Table 8 and Table 9, Figure 6a).

DASH decreased by 4.06 points from baseline to post-therapy, but this change was not statistically significant after Holm correction (Holm-adjusted *p* = 0.228). DASH scores were essentially unchanged from baseline at the 1-month follow-up (mean change = 0.11; adjusted *p* = 0.960) (Table 8 and Table 9, Figure 6b).

## 4. Discussion

This proof-of-concept study examined whether sensor-based RWS could sensitively track longitudinal upper-extremity mobility changes in a cohort of community-dwelling older adults with reduced shoulder ROM receiving a 2-week application of the Spencer Technique.

Total RSA demonstrated the most consistent longitudinal changes. Both dominant and nondominant upper extremity Total RSA increased significantly from baseline to post-therapy and 1-month follow-up, with all four comparisons remaining statistically significant following Holm correction. This sustained increase at 1-month follow-up supports the responsiveness of sensor-derived Total RSA is capable of detecting longitudinal changes in upper-extremity mobility. In the analysis of individual quadrants of Total RSA, the findings were less consistent. Although there were improvements in the upper quadrants (Q1, Q3) at post-therapy and 1-month follow-up and posterior Q5 at 1-month follow-up were seen in both dominant and nondominant arms, only dominant Q1 and nondominant Q5 at 1-month follow-up remained significant after Holm correction. The upper reaching quadrants (Q1, Q3) emphasize improved shoulder flexion and abduction above the shoulder level during active movement against gravity. Individual RSA quadrants highlight changes in the reaching sphere and provide detailed information regarding spatial distribution of RWS but reinforce utility of the Total RSA encapsulating comprehensive upper-extremity reachability.

Shoulder range of motion paralleled changes in Total RSA. There was significant improvement in all shoulder goniometry measurements in flexion and abduction in both dominant and nondominant arms at both post-therapy and 1-month follow-up. All ROM comparisons remained significant after Holm correction. Within-participant effect sizes were moderate to large, with particularly large standardized changes observed for nondominant shoulder flexion and abduction. In our prior cross-sectional study of this population cohort, Total RSA showed moderate-to-strong correlations between goniometric measures of shoulder flexion and abduction and good test–retest reliability [9]. The parallel longitudinal changes observed in goniometer-assessed ROM and Total RSA in the present study further support the responsiveness of Total RSA for detecting changes over time in upper-extremity mobility alongside traditional clinical measures.

The Apley scratch test is a functional range of motion test evaluating active shoulder flexibility, mobility, and coordinated joint movement [22,23]. Both Apley scratch tests showed significant improvement, with decreased inter-finger distance from baseline to post-therapy, with a sustained response during 1-month follow-up. All four planned comparisons remained significant after multiplicity adjustment. These findings paralleled the positive changes in Total RSA, reflecting the ability of sensor-based RWS to capture longitudinal changes in upper-extremity mobility.

NeuroQoL UE and DASH provide patient-reported measures of upper-extremity function and disability, respectively [6,7]. NeuroQoL mean scores increased by 2.0 points at post-therapy and 3.4 points at 1-month follow-up, but only the 1-month follow-up comparison remained significant after Holm correction. The change in mean DASH scores decreased from baseline to post-therapy by 4.06 and was not significant. These self-reported findings were less consistent than the changes observed in the objective measures of goniometric ROM and Total RSA. The objective changes seen in maximal shoulder mobility may not translate into perceived changes in daily activities over the same time period, since many daily activities may not require maximal shoulder ROM. Because this community-dwelling population was selected based on reduced ROM rather than perceived impairment, the discrepancy in the objective findings and patient-reported outcomes may reflect compensatory strategies used to manage restricted shoulder motion, as well as limited room for improvement in perceived upper-extremity function.

The clinical interpretation of RSA becomes more relevant in this population as functional limitations affect ADLs. Early declines in upper-extremity ROM may not immediately produce noticeable functional limitations, and improvements following interventions such as the Spencer Technique may therefore occur before patients perceive a meaningful change in daily function. Importantly, the responsiveness of RSA to subtle changes in multidirectional reach may also provide an opportunity to identify early or subclinical mobility deficits before they become apparent through conventional clinical assessments or patient-reported outcomes. Earlier identification of these changes could support intervention while functional capacity remains relatively preserved, with the goal of maintaining mobility and delaying progression to more overt functional limitations. Although an RSA-specific MCID has not been established, this study emphasizes the ability of Total RSA to be responsive to longitudinal change. This sensor-based assessment offers several potential advantages for longitudinal measurements over traditional clinical assessments. Goniometry measures shoulder ROM in anatomical planes, while Total RSA quantifies a three-dimensional upper extremity reaching sphere integrating movement in multiple directions and planes into a standardized movement pattern that can be quantified and tracked. These qualities support RSA as a complementary quantitative outcome alongside traditional clinical and patient-reported outcomes.

Several limitations should be considered when interpreting this single-group proof-of-concept study. There was a small sample size of 15 participants and the 46 participants used to provide baseline comparison data did not undergo the Spencer Technique or have longitudinal follow-up. The study clinicians and participants could not be blinded. Because there was no control group, the selection based on low ROM risks regression to the mean, practice effects, and natural variation that cannot be excluded as alternate explanations for the observed changes. Although the repeated-measures linear mixed models accounted for within-participant correlation over time, the dominant and nondominant upper extremities were modeled separately, and cross-arm dependence was not explicitly modeled. Holm correction was applied to control for multiple comparisons, but the small sample size limits the precision of the estimates.

Future studies should involve a larger, adequately powered controlled study identifying Total RSA as a primary endpoint. This larger study may establish clinically meaningful change through the examination of RSA and conventional upper-extremity mobility clinical measures and patient-reported outcomes.

## 5. Conclusions

The sensor-based RWS assessment was relatively quick, unobtrusive, and easy to perform in this cohort of older adults. Total RSA demonstrated responsiveness as a quantitative measure of bilateral longitudinal changes in upper-extremity reachability, with parallel changes observed in clinical outcome measures.

## Figures and Tables

**Figure 1:**
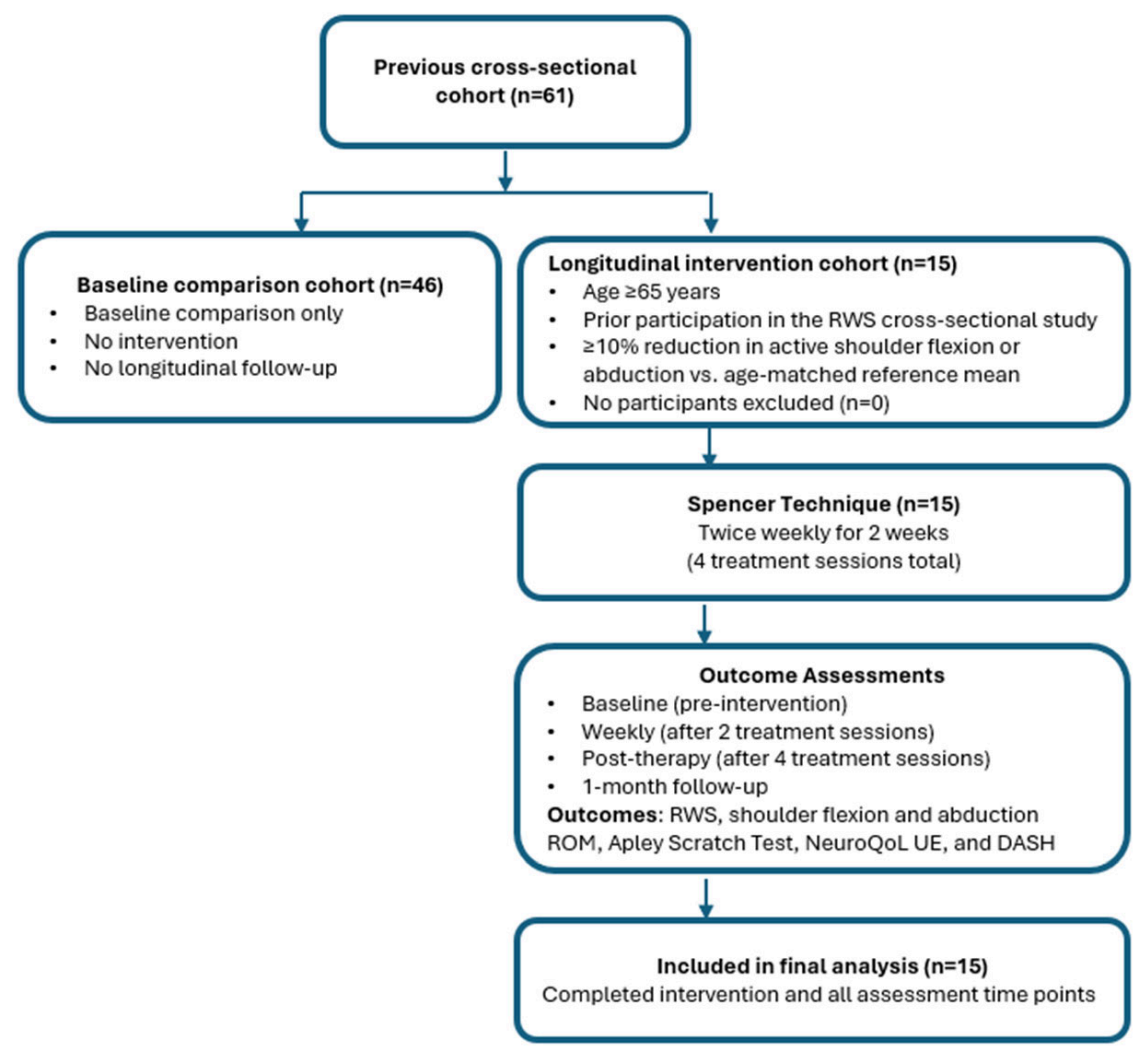
Participant flow and study assessment timeline.

**Figure 2:**
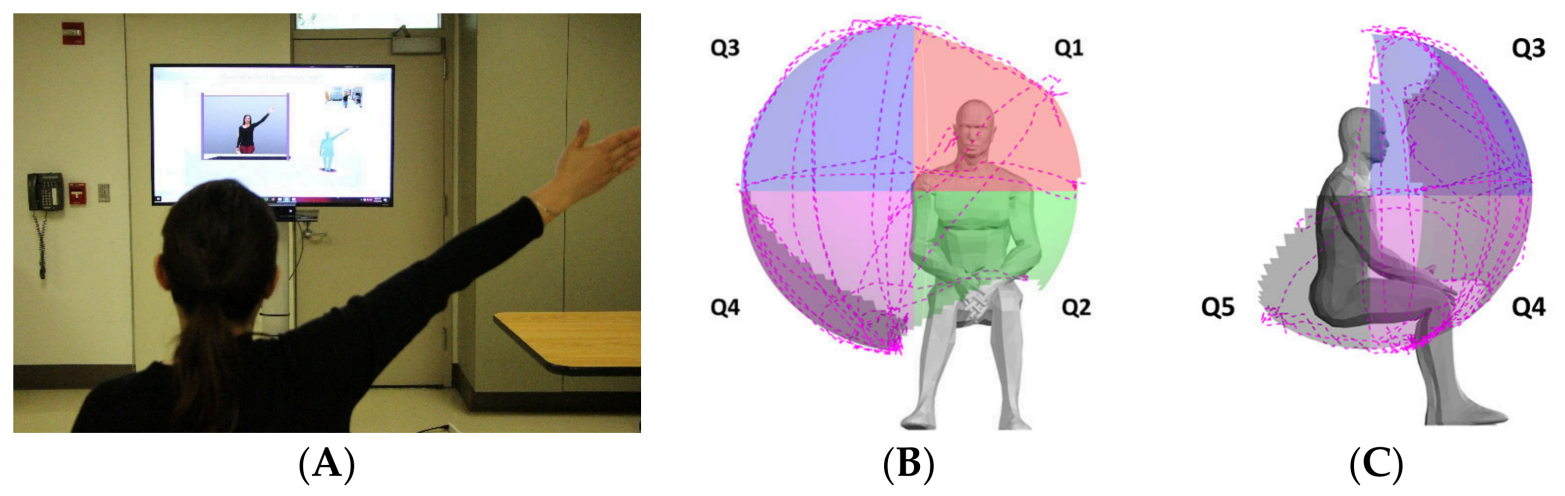
Reachable workspace (RWS) system setup and relative surface area (RSA) envelope output with five quadrants. (**A**) RWS assessment setup. (**B**,**C**) Representative RSA envelope output illustrating the five RWS quadrants (Q1–Q5). The pink dashed lines represent the arm movement trajectories during the RWS assessment. (**B**,**C**) are reprinted from a previous publication [12].

**Figure 3:**
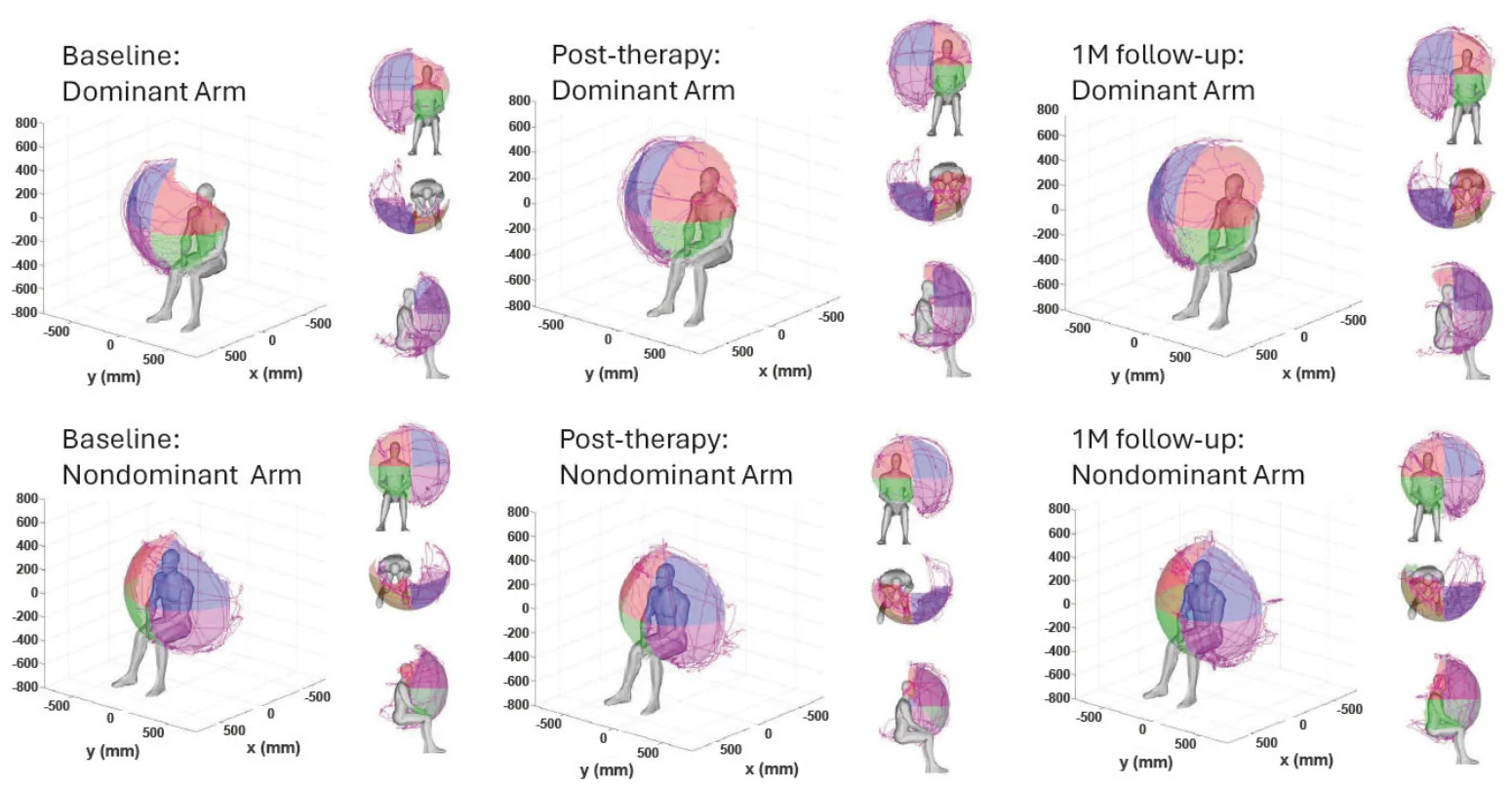
Graphical visualization of the bilateral relative surface area (RSA) of an example study participant at baseline, post-therapy, and 1-month follow-up. The pink dashed lines represent the arm movement trajectories during the RWS assessment.

**Figure 4:**
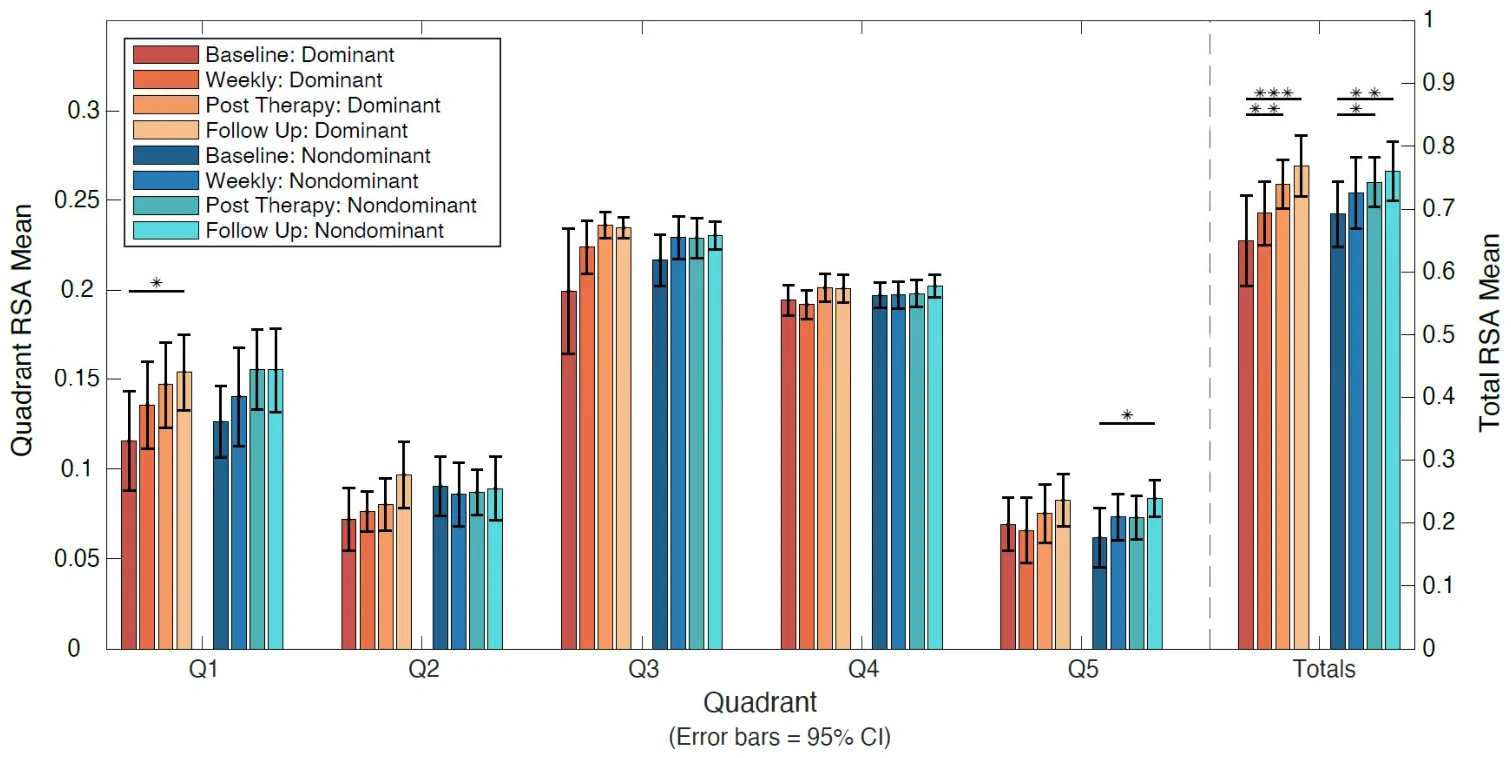
Bar graph of the mean RSAs from the dominant and nondominant arms, showing the individual quadrants’ RSAs (Q1–Q5) and Total RSA at baseline, weekly, post-therapy, and 1-month follow-up. The vertical dashed line separates the individual quadrant RSA values (Q1–Q5) from the total RSA. All evaluations were compared to baseline evaluation. * *p* < 0.05, ** *p* < 0.01, *** *p* < 0.001.

**Figure 5:**
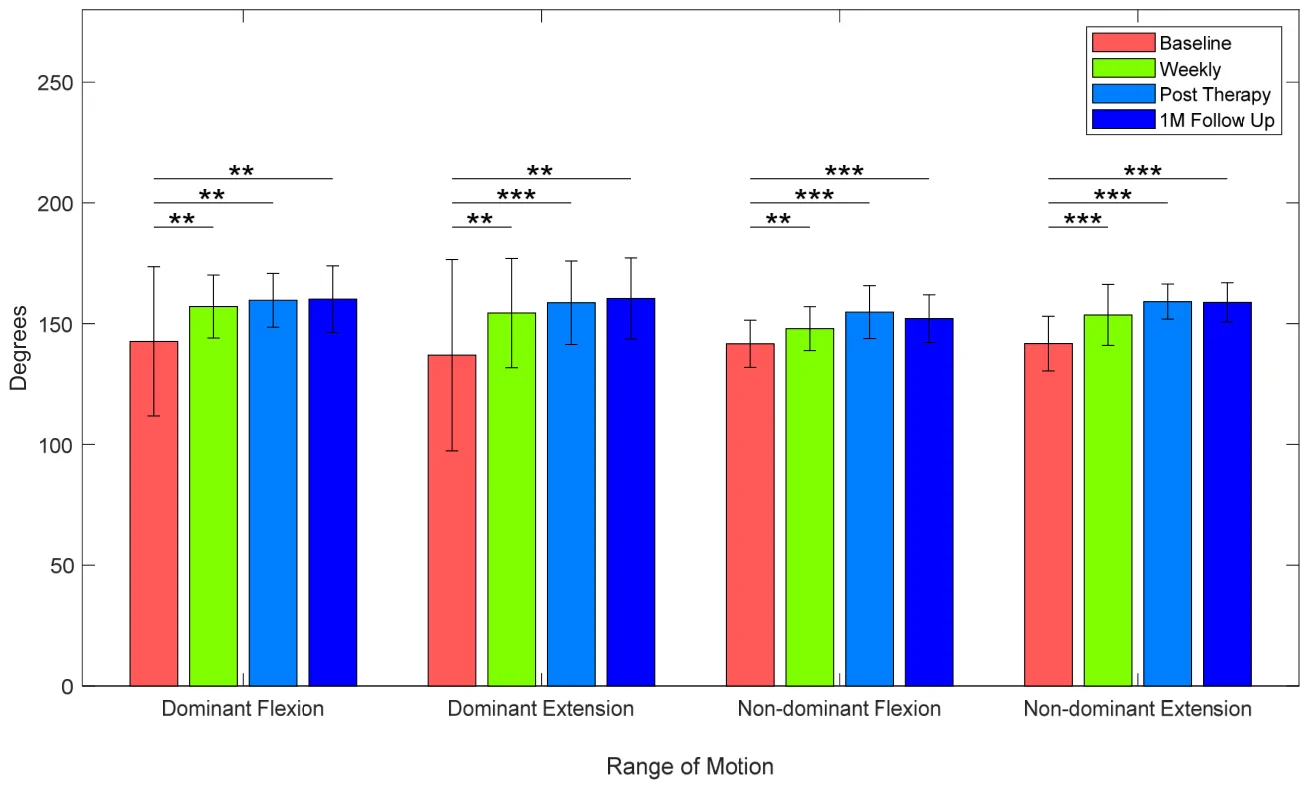
Range of motion (ROM) values for the dominant and nondominant arms at baseline, weekly, post-therapy, and 1-month follow-up evaluations. All evaluations were compared to baseline evaluation. ** *p* < 0.01, *** *p* < 0.001.

**Figure 6:**
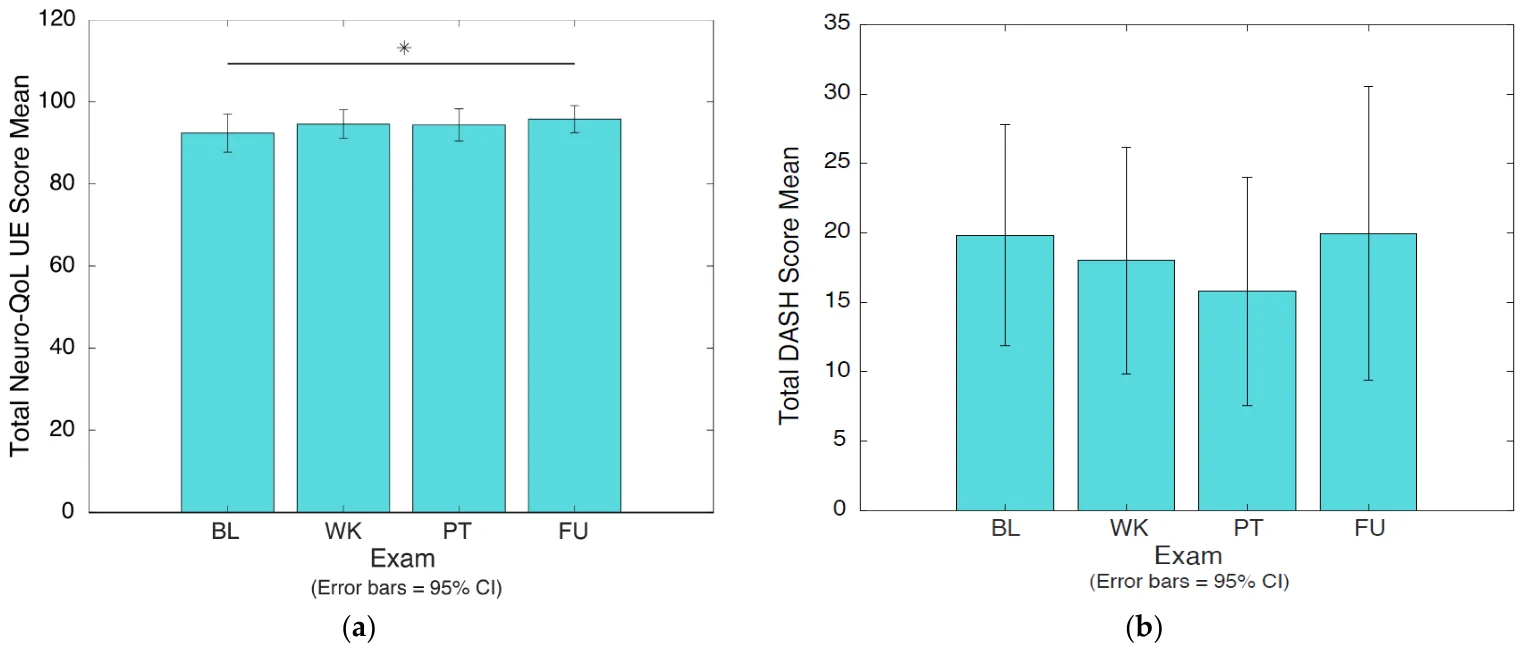
(**a**) NeuroQoL UE mean scores at baseline (BL), weekly (WK), post-therapy (PT), and 1-month follow-up (FU). (**b**) DASH mean scores at baseline (BL), weekly (WK), post-therapy (PT), and 1-month follow-up (FU). All evaluations were compared to the baseline evaluation. * *p* < 0.05.

**Table 1:** Comparison between baseline and study groups.

**Groups**	**Age**	**Height (cm)**	**Weight (kg)**	**DASH**	**UE NeuroQoL**
All (Mean ± SD) *N* = 61	75.20 ± 6.36	165.33 ± 11.09	67.07 ± 16.13	14.26 ± 17.19	96.140 ± 7.28
Baseline (Mean ± SD) *n* = 46	75.09 ± 6.67	164.46 ± 10.37	65.49 ± 14.44	10.96 ± 16.49	97.47 ± 6.65
Study (Mean ± SD) *n* = 15	75.53 ± 5.48	167.98 ± 13.08	71.91 ± 20.30	22.11 ± 15.60	92.77 ± 7.80
*p* value (*t*-test)	0.816	0.290	0.183	0.041 *	0.038 *
**Groups**	**Range of Motion**				
	**D_Flexion**		**D_Abduction**	**ND_Flexion**	**ND_Abduction**
All (Mean ± SD) *N* = 61	160.41 ± 19.90		158.56 ± 23.99	155.30 ± 12.71	154.05 ± 18.37
Baseline (Mean ± SD) *n* = 46	164.89 ± 10.28		164.22 ± 13.90	159.70 ± 9.92	159.33 ± 12.74
Study (Mean ± SD) *n* = 15	146.67 ± 33.00		141.20 ± 37.67	141.8 ± 10.84	137.87 ± 23.53
*p* value (*t*-test)	0.002 **		<0.001 **	<0.001 **	<0.001 **

D, dominant arm; ND, nondominant arm. * *p* < 0.05, ** *p* < 0.01.

**Table 2:** The RSA (mean ± SD) of the dominant and nondominant arms at baseline, weekly, post-therapy, and 1-month follow-up.

**Dominant Arm RSA**	**Baseline**	**Weekly**	**Post-Therapy**	**1-Month Follow-Up**
Quadrant 1	0.115 ± 0.055	0.135 ± 0.048	0.147 ± 0.048	0.154 ± 0.042
Quadrant 2	0.072 ± 0.035	0.076 ± 0.022	0.080 ± 0.029	0.097 ± 0.036
Quadrant 3	0.199 ± 0.069	0.224 ± 0.029	0.236 ± 0.015	0.235 ± 0.011
Quadrant 4	0.194 ± 0.017	0.192 ± 0.016	0.201 ± 0.016	0.201 ± 0.015
Quadrant 5	0.069 ± 0.029	0.066 ± 0.036	0.075 ± 0.032	0.083 ± 0.029
Total (Q1–Q5)	0.650 ± 0.141	0.693 ± 0.098	0.740 ± 0.075	0.769 ± 0.096
**Nondominant Arm RSA**	**Baseline**	**Weekly**	**Post-Therapy**	**1-Month Follow-Up**
Quadrant 1	0.126 ± 0.039	0.140 ± 0.055	0.155 ± 0.045	0.155 ± 0.046
Quadrant 2	0.090 ± 0.032	0.086 ± 0.035	0.087 ± 0.025	0.089 ± 0.035
Quadrant 3	0.217 ± 0.029	0.229 ± 0.024	0.229 ± 0.022	0.230 ± 0.015
Quadrant 4	0.197 ± 0.014	0.197 ± 0.015	0.198 ± 0.015	0.202 ± 0.012
Quadrant 5	0.062 ± 0.032	0.073 ± 0.025	0.073 ± 0.024	0.084 ± 0.020
Total (Q1–Q5)	0.692 ± 0.101	0.726 ± 0.112	0.743 ± 0.077	0.760 ± 0.093

**Table 3:** Linear mixed-model analysis of Total RSA of the dominant and nondominant arms.

Outcome	Comparison	Mean Change (95% CI)	Raw *p*	Holm-Adjusted *p*	Cohen’s *dz*	Overall Time Effect
Dominant Total RSA	Post-therapy	0.090 (0.036–0.143)	0.002	0.005 **	0.74	F(3,42) = 7.62, *p* < 0.001
	1-month follow-up	0.119 (0.065–0.172)	<0.001	<0.001 ***	0.88	
Nondominant Total RSA	Post-therapy	0.050 (0.005–0.096)	0.03	0.030 *	0.79	F(3,42) = 3.35, *p* = 0.028
	1-month follow-up	0.068 (0.023–0.114)	0.004	0.008 **	0.65	

All evaluations were compared to baseline evaluation. * *p* < 0.05, ** *p* < 0.01, *** *p* < 0.001.

**Table 4:** Shoulder flexion and shoulder abduction range of motion (mean ± SD) of the dominant and nondominant upper extremities at baseline, weekly, post-therapy, and 1-month follow-up.

	Baseline	Weekly	Post-Therapy	1-Month Follow-Up
Dominant Arm Flexion	142.670 ± 30.891	157.067 ± 13.046	159.670 ± 11.133	160.130 ± 13.799
Dominant Arm Abduction	136.930 ± 39.590	154.400 ± 22.617	158.670 ± 17.319	160.470 ± 16.758
Nondominant Arm Flexion	141.670 ± 9.766	147.933 ± 9.106	154.800 ± 10.962	152.070 ± 9.859
Nondominant Arm Abduction	141.730 ± 11.298	153.600 ± 12.642	159.130 ± 7.279	158.800 ± 8.126

**Table 5:** Linear mixed-model analysis of shoulder range of motion.

Outcome	Comparison	Mean Change (95% CI), °	Raw *p*	Holm-Adjusted *p*	Cohen’s *dz*	Overall Time Effect
Dominant flexion	Post-therapy	17.0 (8.0–26.0)	<0.001	<0.001 ***	0.72	F (3,42) = 6.91, *p* < 0.001
	1-month follow-up	17.5 (8.5–26.4)	<0.001	<0.001 ***	0.7	
Dominant Abduction	Post-therapy	21.7 (11.9–31.6)	<0.001	<0.001 ***	0.9	F (3,42) = 9.78, *p* < 0.001
	1-month follow-up	23.5 (13.7–33.4)	<0.001	<0.001 ***	0.82	
Nondominant flexion	Post-therapy	13.1 (8.2–18.0)	<0.001	<0.001 ***	1.17	F (3,42) = 11.07, *p* < 0.001
	1-month follow-up	10.4 (5.5–15.3)	<0.001	<0.001 ***	0.96	
Nondominant abduction	Post-therapy	17.4 (11.8–23.0)	<0.001	<0.001 ***	2.14	F (3,42) = 17.14, *p* < 0.001
	1-month follow-up	17.1 (11.5–22.7)	<0.001	<0.001 ***	1.33	

All evaluations were compared to baseline evaluation. *** *p* < 0.001.

**Table 6:** The mean and standard deviations of the Apley 1 and Apley 2 movements at baseline, weekly, post-therapy, and 1-month follow-up.

	Baseline	Weekly	Post-Therapy	1-Month Follow-Up
Apley 1 (cm)	23.589 ± 11.571	20.922 ± 11.726	19.089 ± 11.177	19.111 ± 9.966
Apley 2 (cm)	16.722 ± 12.620	14.467 ± 13.417	12.033 ± 12.041	11.433 ± 10.484

**Table 7:** Linear mixed-model analysis of Apley measures of the dominant and nondominant arms.

Outcome	Comparison	Mean Change (95% CI)	Raw *p*	Holm-Adjusted *p*	Cohen’s *dz*	Overall Time Effect
Apley 1	Post-therapy	−4.50 (−6.12 to −2.88)	<0.001	<0.001 ***	−1.71	F (3,42) = 13.94, *p* < 0.001
	1-month follow-up	−4.48 (−6.10 to −2.85)	<0.001	<0.001 ***	−1.17	
Apley 2	Post-therapy	−4.69 (−6.60 to −2.77)	<0.001	<0.001 ***	−1.28	F (3,42) = 13.03, *p* < 0.001
	1-month follow-up	−5.29 (−7.20 to −3.37)	<0.001	<0.001 ***	−1.07	

Apley 1 = ND Flexion, ER & Abd; D Extension, IR & Add. Apley 2 = D Flexion, ER & Abd; ND Extension, IR & Add. All evaluations were compared to baseline evaluation. *** *p* < 0.001.

**Table 8:** NeuroQoL UE and DASH scores (mean ± SD) at baseline, weekly, post-therapy, and 1-month follow-up.

	Baseline	Weekly	Post-Therapy	1-Month Follow-Up
NeuroQoL UE	92.400 ± 8.716	94.600 ± 6.512	94.400 ± 7.327	95.800 ± 6.190
(Min, Max)	(72, 100)	(75, 100)	(71, 100)	(75, 100)
DASH	19.833 ± 14.928	18.000 ± 15.223	15.778 ± 15.403	19.944 ± 19.762
(Min, Max)	(1.667, 55.000)	(0.000, 56.667)	(0.833, 60.833)	(1.667, 69.167)

**Table 9:** Linear mixed-model analysis of patient-reported outcomes.

Outcome	Comparison	Mean Change (95% CI)	Raw *p*	Holm-Adjusted *p*	Cohen’s *dz*	Overall Time Effect
NeuroQoL UE	Post-therapy	2.00 (−0.45 to 4.45)	0.107	0.228	0.35	F (3,42) = 2.69, *p* = 0.059
	1-month follow-up	3.40 (0.95 to 5.85)	0.008	0.031 *	0.49	
DASH	Post-therapy	−4.06 (−8.55 to 0.44)	0.076	0.228	−0.53	F (3,42) = 1.54, *p* = 0.218
	1-month follow-up	0.11 (−4.39 to 4.61)	0.96	0.96	0.01	

All evaluations were compared to baseline evaluation. * *p* < 0.05.

## Data Availability

The data sets generated during and/or analyzed during the current study are available from the corresponding author upon reasonable request.

## Supplementary Materials

The following supporting information can be downloaded at https://www.mdpi.com/article/10.3390/s26196305/s1. Table S1: Quadrant-Specific Relative Surface Area (RSA): Planned Contrasts and Overall Time Effects for the Dominant and Nondominant Upper Extremities.

## Abbreviations

ADL	Activity of Daily Living
ABD	Abduction
ADD	Adduction
DASH	Disabilities of the Arm, Shoulder, and Hand
ER	External Rotation
IR	Internal Rotation
IRB	Institutional Review Board
MCID	Minimal Clinically Important Difference
PRO	Patient-Reported Outcomes
ROM	Range of Motion
RSA	Relative Surface Area
