# Supplementary material for: Sensor-Based Reachable Workspace Assessment for Tracking Upper-Extremity Mobility Changes over Time Following the Spencer Technique in Older Adults
Source: Sensors (Basel). 2026 Oct 6;26(19):6305. doi: 10.3390/s26196305 (PMC13667728; doi:10.3390/s26196305)
Supplement: Supplementary file 1 [file sensors-26-06305-s001.zip › sensors-4564797-supplementary.pdf]

Supplementary Table S1. Quadrant-Specific Relative Surface Area (RSA): Planned Contrasts and Overall Time Effects for the Dominant and Nondominant Upper Extremities.

Outcome	Comparison	Mean change (95% CI)	Raw *p*	Holm-adjusted *p*	Cohen's *dz*	Overall Time Effect
Dominant Q1	Post-therapy	0.031 (0.007–0.055)	0.012	0.128	0.54	F (3,42) = 3.97, p = .014
	1-month follow-up	0.038 (0.014–0.062)	0.002	.046*	0.68	
Dominant Q2	Post-therapy	0.008 (−0.009–0.025)	0.328	1	0.24	F (3,42) = 3.33, p = .028
	1-month follow-up	0.025 (0.008–0.042)	0.005	0.081	0.89	
Dominant Q3	Post-therapy	0.037 (0.013–0.061)	0.004	0.064	0.58	F (3,42) = 4.03, p = .013
	1-month follow-up	0.035 (0.011–0.059)	0.005	0.081	0.52	
Dominant Q4	Post-therapy	0.007 (−0.002–0.016)	0.135	1	0.37	F (3,42) = 2.12, p = .112
	1-month follow-up	0.007 (−0.003–0.016)	0.157	1	0.35	
Dominant Q5	Post-therapy	0.006 (−0.007–0.020)	0.359	1	0.31	F (3,42) = 2.47, p = .075
	1-month follow-up	0.013 (−0.000–0.027)	0.05	0.502	0.81	
Nondominant Q1	Post-therapy	0.029 (0.009–0.050)	0.006	0.089	0.91	F (3,42) = 3.77, p = .018
	1-month follow-up	0.029 (0.008–0.049)	0.007	0.089	0.65	
Nondominant Q2	Post-therapy	−0.003 (−0.022–0.015)	0.708	1	−0.11	F (3,42) = 0.10, p = .959
	1-month follow-up	−0.001 (−0.020–0.017)	0.882	1	−0.03	
Nondominant Q3	Post-therapy	0.013 (0.003–0.022)	0.008	0.102	0.75	F (3,42) = 4.18, p = .011
	1-month follow-up	0.014 (0.005–0.023)	0.004	0.064	0.62	
Nondominant Q4	Post-therapy	0.001 (−0.006–0.008)	0.756	1	0.08	F (3,42) = 0.91, p = .446
	1-month follow-up	0.005 (−0.002–0.012)	0.156	1	0.33	
Nondominant Q5	Post-therapy	0.011 (−0.001–0.023)	0.068	0.616	0.49	F (3,42) = 4.50, p = .008
	1-month follow-up	0.022 (0.010–0.034)	<.001	.013*	0.67	

All evaluations were compared to Baseline Evaluation.
*$p<0.05$
